# Targeting novel regulated cell death: disulfidptosis in cancer immunotherapy with immune checkpoint inhibitors

**DOI:** 10.1186/s40364-025-00748-4

**Published:** 2025-02-26

**Authors:** Fei Du, Guojun Wang, Qian Dai, Jiang Huang, Junxin Li, Congxing Liu, Ke Du, Hua Tian, Qiwei Deng, Longxiang Xie, Xin Zhao, Qimin Zhang, Lan Yang, Yaling Li, Zhigui Wu, Zhuo Zhang

**Affiliations:** 1 Department of Pharmacy, The Fourth Affiliated Hospital Of Southwest Medical University, Meishan, 620000 Sichuan China; 2 Department of Pharmacology, School of Pharmacy, Southwest Medical University, Luzhou, 646000 Sichuan China; 3 Department of Pharmacy, The Affiliated Traditional Chinese Medicine Hospital of Southwest Medical University, Luzhou, 646000 Sichuan China; 4 Department of pharmacy, Zigong Fourth People’s Hospital, Zigong, 643000 China; 5 Department of Pharmacy, Chengfei Hospital, Chengdu, 610000 China; 6 Department of Pediatrics, Luzhou Maternal and Child Health Hospital, Luzhou Second People’s Hospital, Luzhou, 646000 Sichuan China; 7 School of Nursing, Chongqing College of Humanities, Science & Technology, Chongqing, 401520 China; 8 Heruida Pharmaceutical Co.,ltd, Haikou, Hainan, 570100 China; 9 The TCM Hospital of Longquanyi District, Chengdu, 610100 Sichuan China; 10 Department of Pharmacy, The Affiliated Hospital of Southwest Medical University, Luzhou, 646000 Sichuan China

**Keywords:** Disulfidptosis, Immune checkpoint inhibitors, Regulatory cell death, SLC7A11, Immunotherapy

## Abstract

The battle against cancer has evolved over centuries, from the early stages of surgical resection to contemporary treatments including chemotherapy, radiation, targeted therapies, and immunotherapies. Despite significant advances in cancer treatment over recent decades, these therapies remain limited by various challenges. Immune checkpoint inhibitors (ICIs), a cornerstone of tumor immunotherapy, have emerged as one of the most promising advancements in cancer treatment. Although ICIs, such as CTLA-4 and PD-1/PD-L1 inhibitors, have demonstrated clinical efficacy, their therapeutic impact remains suboptimal due to patient-specific variability and tumor immune resistance. Cell death is a fundamental process for maintaining tissue homeostasis and function. Recent research highlights that the combination of induced regulatory cell death (RCD) and ICIs can substantially enhance anti-tumor responses across multiple cancer types. In cells exhibiting high levels of recombinant solute carrier family 7 member 11 (SLC7A11) protein, glucose deprivation triggers a programmed cell death (PCD) pathway characterized by disulfide bond formation and REDOX (reduction-oxidation) reactions, termed “disulfidptosis.” Studies suggest that disulfidptosis plays a critical role in the therapeutic efficacy of SLC7A11^high^ cancers. Therefore, to investigate the potential synergy between disulfidptosis and ICIs, this study will explore the mechanisms of both processes in tumor progression, with the goal of enhancing the anti-tumor immune response of ICIs by targeting the intracellular disulfidptosis pathway.

## Interaction

ICIs represent a significant breakthrough in cancer immunotherapy in recent years. Immune checkpoints are crucial in regulating immune system activity and balance, with early research on immune checkpoints dating back to the late 20th century [1, 2]. In 1987, Pierre Golstein and colleagues first identified CTLA-4 (cytotoxic T lymphocyte antigen 4) [3]. In 1995, Tak Wah Mak and Arlene H. Sharpe’s research using CTLA-4 gene knockout mice revealed its role as a negative regulator of T cell activation [4, 5]. PD-1 (programmed cell death protein 1) was identified by Professor Tasuku Honjo in the early 1990s, who later proposed that PD-1 serves as a negative regulator of the immune system in 1999 [6, 7].

Since the Food and Drug Administration (FDA)’s approval of ICIs in 2011, these therapies have revolutionized cancer treatment. Monoclonal antibodies targeting immune checkpoints have demonstrated clinical efficacy in liver cancer, non-small cell lung cancer, colorectal cancer, melanoma, and other malignancies [8, 9]. However, while some patients exhibit long-term responses, the objective response rate (ORR) for ICI monotherapy remains relatively low, with response rates of only 10–20% in second-line therapies and beyond [10, 11]. This suggests the involvement of other immune-related pathways in immunopathogenesis and therapeutic resistance. To overcome the limitations of ICIs in tumor immunotherapy, such as low ORR, immune-related adverse events (irAEs), and therapeutic resistance, it is crucial to develop strategies that address the multifaceted complexities of the tumor microenvironment (TME). Currently, researchers are focusing on targeting immunogenic cell death (ICD) alongside ICI therapy in the TME to enhance immunotherapy by reactivating T cells and improving patient responses to ICIs [12, 13].

Cell death is a vital homeostatic mechanism that maintains tissue morphology and function in vivo. In 2018, the Nomenclature Committee on Cell Death (NCCD) officially categorized cell death into accidental cell death (ACD) and RCD [14]. ACD is primarily induced by external factors, such as physical or chemical damage, and occurs without cellular regulation [15], whereas RCD is controlled by a series of intracellular signaling pathways, resulting in distinct biological processes, including apoptosis, pyroptosis, ferroptosis, and cuproptosis [15, 16]. In the 1980s, researchers observed that certain chemicals, such as chlorinated benzene and sulfoxide chloride, led to the production of disulfide bond molecules within cells, ultimately causing cell death [17, 18]. However, the specific mechanism underlying disulfide bond-induced cell death remained unclear until 2020, when Professor Boyi Gan’s research team discovered that, under glucose scarcity, SLC7A11^high^ cells rapidly deplete nicotinamide adenine dinucleotide phosphate (NADPH), leading to the abnormal accumulation of cystine and other disulfides. This results in a distinct cell death pattern, independent of ferroptosis or apoptosis, and highly dependent on NADPH generated by the pentose phosphate pathway (PPP) and the uptake and conversion of cystine mediated by SLC7A11, a process termed “disulfidptosis“ [19]. Increasing evidence links disulfidptosis to the pathogenesis of various diseases, including cancer, inflammation, and cardiovascular disease [20, 21]. Consequently, targeting disulfidptosis in the TME may emerge as a promising strategy for cancer treatment.

**Fig. 1:**
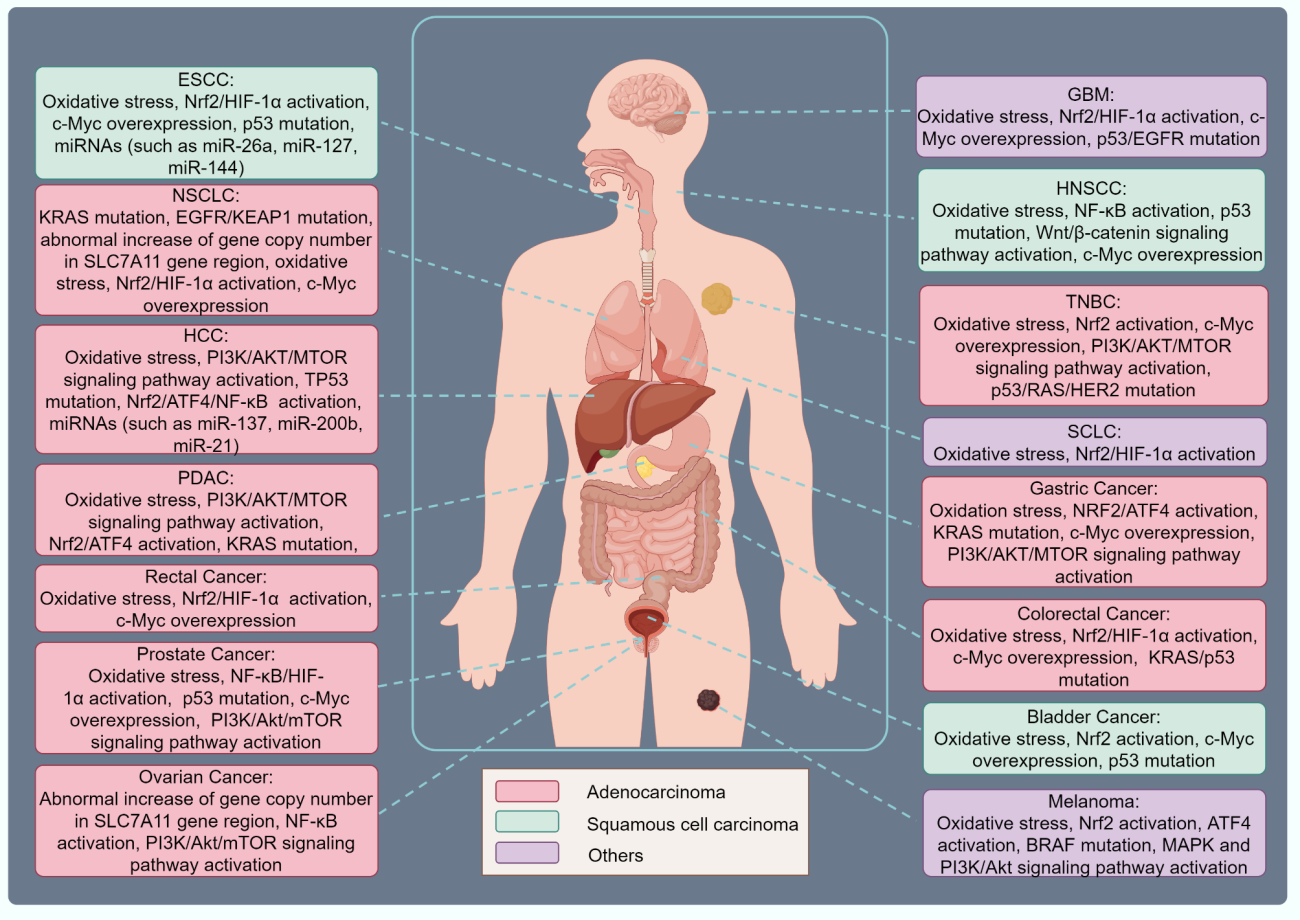
Tumors with high expression of SLC7A11 and its mechanism. Different activation or mutation mechanisms drive the overexpression of SLC7A11 in tumor cells across various anatomical locations. NSCLC, non-small cell lung carcinoma; SCLC, small cell lung carcinoma; GBM, glioblastoma multiforme; ESCC, esophageal squamous cell carcinoma; HCC, hepatocellular carcinoma; TNBC, triple negative breast cancer; PDAC, pancreatic ductal adenocarcinoma; HNSCC, head and neck squamous cell carcinoma; KRAS, kirsten rat sarcoma viral oncogene homolog; Nrf2, nuclear factor erythroid 2-related factor 2; HIF-1α, hypoxia-inducible factor 1-alpha; SLC7A11, solute carrier family 7 member 11; c-Myc, cellular myelocytomatosis oncogene; MAPK, mitogen-activated protein kinase; BRAF, B-Raf proto-oncogene, serine/threonine kinase; SP1, specificity protein 1; KEAP1, kelch-like ECH-associated protein 1; ATF4, activating transcription factor 4; NF-κB, nuclear factor kappa-light-chain-enhancer of activated B cells

SLC7A11 (also known as xCT) is a key member of the solute carrier family and functions as an essential amino acid transporter [22]. By facilitating cysteine uptake and glutamate release, SLC7A11 promotes the synthesis of glutathione (GSH), maintains cellular REDOX balance, and prevents lipid peroxidation (LPO)-induced cell death [22, 23]. Recent studies have shown that SLC7A11 is highly expressed in several tumor types, including lung cancer, breast cancer, colorectal cancer, and melanoma, and plays a pivotal role in the immune evasion mechanisms of tumors [24, 25] (Table 1) (Fig. 1). Additionally, high SLC7A11 expression has been shown to promote the accumulation of immunosuppressive cells, such as myeloid-derived suppressor cells (MDSCs), within the TME, further inhibiting effector T cell activity and diminishing the efficacy of anti-tumor immune responses [26, 27]. These observations offer new insights into tumor immune escape mechanisms and suggest potential targets for immunotherapy.

**Table 1:** Tumors with high expression of SLC7A11 and its mechanism

Type	Tumor	Mechanism	Reference
**Adenocarcinoma**	NSCLC	KRAS mutation, EGFR mutation/activation, abnormal increase of gene copy number in SLC7A11 gene region, oxidative stress, HIF-1α activation, Nrf2 activation, c-Myc overexpression, KEAP1 mutation	[28, 29]
	HCC	Oxidative stress, PI3K/AKT/MTOR signaling pathway activation, TP53 mutation, Nrf2 activation, ATF4 activation, miRNAs (such as miR-137, miR-200b, miR-21), NF-κB activation	[22, 30, 31]
	TNBC	Oxidative stress, Nrf2 activation, c-Myc overexpression, PI3K/AKT/MTOR signaling pathway activation, cancer driver genes (such as p53, RAS, HER2) mutations	[32–34]
	PDAC	Oxidative stress, PI3K/AKT/MTOR signaling pathway activation, Nrf2 activation, KRAS mutation, ATF4 activation	[35–37]
	Colorectal Cancer	Oxidative stress, Nrf2 activation, c-Myc overexpression, HIF-1αactivation, KRAS mutation, p53 mutation	[38–40]
	Rectal Cancer	Oxidative stress, Nrf2 activation, c-Myc overexpression, HIF-1αactivation	[41]
	Gastric Cancer	Oxidation stress, NRF2 activation, KRAS mutation, c-Myc overexpression, PI3K/AKT/MTOR signaling pathway activation, ATF4 activation	[42–44]
	Prostate Cancer	Oxidative stress, NF-κB activation, HIF-1α activation, p53 mutation, c-Myc overexpression, and PI3K/Akt/mTOR signaling pathway activation	[45–47]
	Ovarian Cancer	abnormal increase of gene copy number in SLC7A11 gene region, NF-κB activation, PI3K/Akt/mTOR signaling pathway activation	[47–49]
**Squamous cell carcinoma**	Bladder Cancer	Oxidative stress, Nrf2 activation, c-Myc overexpression, p53 mutation	[50, 51]
	HNSCC	Oxidative stress, NF-κB activation, p53 mutation, Wnt/β-catenin signaling pathway activation, c-Myc overexpression	[52–54]
	ESCC	Oxidative stress, Nrf2 activation, c-Myc overexpression, p53 mutation, HIF-1αactivation, miRNAs (such as miR-26a, miR-127, miR-144)	[52, 55]
**Others**	SCLC	Oxidative stress, Nrf2 activation, HIF-1αactivation	[56]
	Melanoma	Oxidative stress, Nrf2 activation, ATF4 activation, BRAF mutation, MAPK and PI3K/Akt signaling pathway activation	[57–59]
	GBM	Oxidative stress, Nrf2 activation, c-Myc overexpression, p53 mutation, EGFR mutation, HIF-1αactivation, PI3K/AKT/MTOR signaling pathway activation	[60–62]

NSCLC, non-small cell lung carcinoma; SCLC, non-small cell lung carcinoma; GBM, glioblastoma multiforme; ESCC, esophageal squamous cell carcinoma; HCC, hepatocellular carcinoma; TNBC, triple negative breast cancer; PDAC, pancreatic ductal adenocarcinoma; HNSCC, head and neck squamous cell carcinoma; KRAS, kirsten rat sarcoma viral oncogene homolog; Nrf2, nuclear factor erythroid 2-related factor 2; HIF-1α, hypoxia-inducible factor 1-alpha; SLC7A11, solute carrier family 7 member 11; c-Myc, cellular myelocytomatosis oncogene; MAPK, mitogen-activated protein kinase; BRAF, B-Raf proto-oncogene, serine/threonine kinase; SP1, specificity protein 1; KEAP1, kelch-like ECH-associated protein 1; ATF4, activating transcription factor 4; NF-κB, nuclear factor kappa-light-chain-enhancer of activated B cells

This review synthesizes current research on ICIs and disulfidptosis in cancer immunotherapy, exploring the regulatory relationships between disulfidptosis-related pathways, genes, and immune checkpoints. The aim is to investigate the potential of combining disulfidptosis targeting with ICIs, providing a novel therapeutic strategy for treating SLC7A11^high^ tumors.

## ICIs

In recent years, immunotherapy has demonstrated substantial clinical efficacy against various cancer types. Among the diverse therapeutic strategies, ICIs have emerged as the most widely utilized form of cancer immunotherapy in clinical practice. Although only a subset of patients currently exhibits clinical responses, ICIs have nevertheless revolutionized cancer treatment for many individuals [2, 8]. ICIs primarily counteract the immunosuppressive effects of tumor cells on T cells by blocking the interaction between immunosuppressive regulatory proteins on the cell surface, such as CTLA-4, PD-1, and PD-L1. This mechanism restores or enhances the cytotoxic activity of T cells against tumors [63]. Specifically, ICIs targeting CTLA-4 and PD-1/PD-L1 have become the focal point of recent tumor immunotherapy research (Table 2).

**Table 2:** Research status of immune checkpoint

Type	Target	Drug	Indication	Reference
**Inhibitory molecule**	CTLA-4	Abatacept	DMARDs	[64]
		Lpilimumab	Melanoma, RCC	[65]
		Belatacept	Prevention of transplant rejection in adult kidney transplant recipients	[66, 67]
		Tremelimumab-actl	HCC, NSCLC	[68]
		Cadonilimab	Solid tumor	[69]
	PD-1	Nivolumab	NSCLC, Melanoma, RCC, HL	[70, 71]
		Pembrolizumab	Melanoma, NSCLC, HL, RCC, HCC, HNCA, UTUC, Gastric CA, Esophageal CA, TNBC, MSI-H, CSCC	[72–75]
		Cemiplimab	CSCC, NSCLC	[76]
		Dostarlimab	MSI-H or dMMR tumors	[77, 78]
		Toripalimab	NPC, NSCLC	[79, 80]
		Sintilimab	HL, NSCLC, HCC, Esophageal CA, GEJ Adenocarcinoma	[81, 82]
		Camrelizumab	HL, NSCLC, HCC, Esophageal CA, NPC	[83–85]
		Tislelizumab	HL, NSCLC, HCC, Esophageal CA, NPC, MSI-H or dMMR tumors, UC, CSCC	[86–88]
		Zimberelimab	CC, HL	[89]
	PD-L1	Atezolizumab	NSCLC, TNBC, Bladder tumor	[90, 91]
		Durvalumab	UC, NSCLC	[92, 93]
		Avelumab	MCC, UC	[94, 95]
		Sugemalimab	Lymphoma, Esophageal CA, NSCLC	[96, 97]
		Socazolimab	CC, MCC, ESCC	[98, 99]
	LAG-3	Relatlimab	Melanoma, NSCLC, CRC	[70, 100, 101]
		SHR-1802	Solid tumor	[102]
		ZGGS15	CRC	[103]
		ABL501	CCA	[104]
		Favezelimab	CRC, NSCLC	[105]
	TIM-3	Sabatolimab	CMML, MDS, GBM	[106, 107]
		Cobolimab	Melanoma, NSCLC, HCC	[108]
		LY3321367	Solid tumor	[109]
	TIGIT	Etigilimab	NSCLC, HNCA	[110]
		Vibostolimab	Melanoma, NSCLC	[110, 111]
		Domvanalimab	SCLC, HNCA	[112]
	VISTA	Tiragolumab	NSCLC, ESCC	[113]
		Ociperlimab	Solid tumor	[114]
	BTLA	Icatolimab	Solid tumor	[115]
		Tifcemalimab	Solid tumor	[115]
	CD47	Ligufalimab	Solid tumor	[116]
		Hu5F9-G4	OC, FTC	[117]
		Evorpacept	GEJ Adenocarcinoma	[118]
**Costimulatory molecule**	CD27	Varlilumab	Solid tumor	[119]
	OX40	MEDI6383	Melanoma, Lymphoma	[120]
		MOXR0916	Solid tumor	[121]
	4-1BB	Urelumab	Melanoma, Lymphoma, RCC	[122, 123]
		Utomilumab	Solid tumor	[124]
	GITR	TRX518	Solid tumor	[125]
		MEDI1873	Solid tumor	[126]

CTLA-4, cytotoxic t-lymphocyte antigen 4; PD-1, programmed cell death protein 1; PD-L1, programmed cell death ligand 1; LAG-3, lymphocyte activation gene 3; TIM-3, T-cell immunoglobulin and mucin domain 3; TIGIT, T-cell immunoreceptor with Ig and ITIM domain; VISTA, V-domain ig suppressor of T cell activation; BTLA, B and T lymphocyte attenuator; OX40, oxygen-40; 4-1BB, tumor necrosis factor receptor superfamily member 9; GITR; glucocorticoid-induced tnfr-related gene; DMARDs, disease-modifying anti-rheumatic drugs; RCC, renal cell carcinoma; HCC, hepatocellular carcinoma; NSCLC, non-small cell lung carcinoma; SCLC, non-small cell lung carcinoma; HL, hodgkin lymphoma; HNCA, head and neck cancer; UTUC, upper tract urothelial carcinoma; CC, cervical cancer; Gastric CA, gastric carcinoma; Esophageal CA, esophageal carcinoma; CSCC, cutaneous squamous-cell carcinoma; MSI-H, high microsatellite instability; dMMR, mismatch repair defect; MCC, merkel cell carcinoma; NPC; nasopharyngeal carcinoma; TNBC, triple negative breast cancer; UC, urothelial carcinoma; CRC, colorectal cancer; CCA, cholangiocarcinoma; CMML, chronic myelomonocytic leukemia; MDS, myelodysplastic syndromes; GBM, glioblastoma multiforme; ESCC, esophageal squamous cell carcinoma; OC, ovarian cancer; FTC, fallopian tube cancer; GEJ Adenocarcinoma, gastric or gastroesophageal junction adenocarcinoma;

### CTLA-4 inhibitors

The discovery of CTLA-4 dates back to the 1990s [3]. Early animal studies revealed that CTLA-4 deficiency could lead to autoimmune disease, suggesting its pivotal role in immune regulation [4, 5]. In the early 2000s, Ipilimumab, the first CTLA-4 inhibitor, entered clinical trials and demonstrated remarkable efficacy in patients with melanoma [127]. In 2011, the FDA approved Ipilimumab, marking the first immune checkpoint inhibitor approved for cancer immunotherapy [128].

**Fig. 2:**
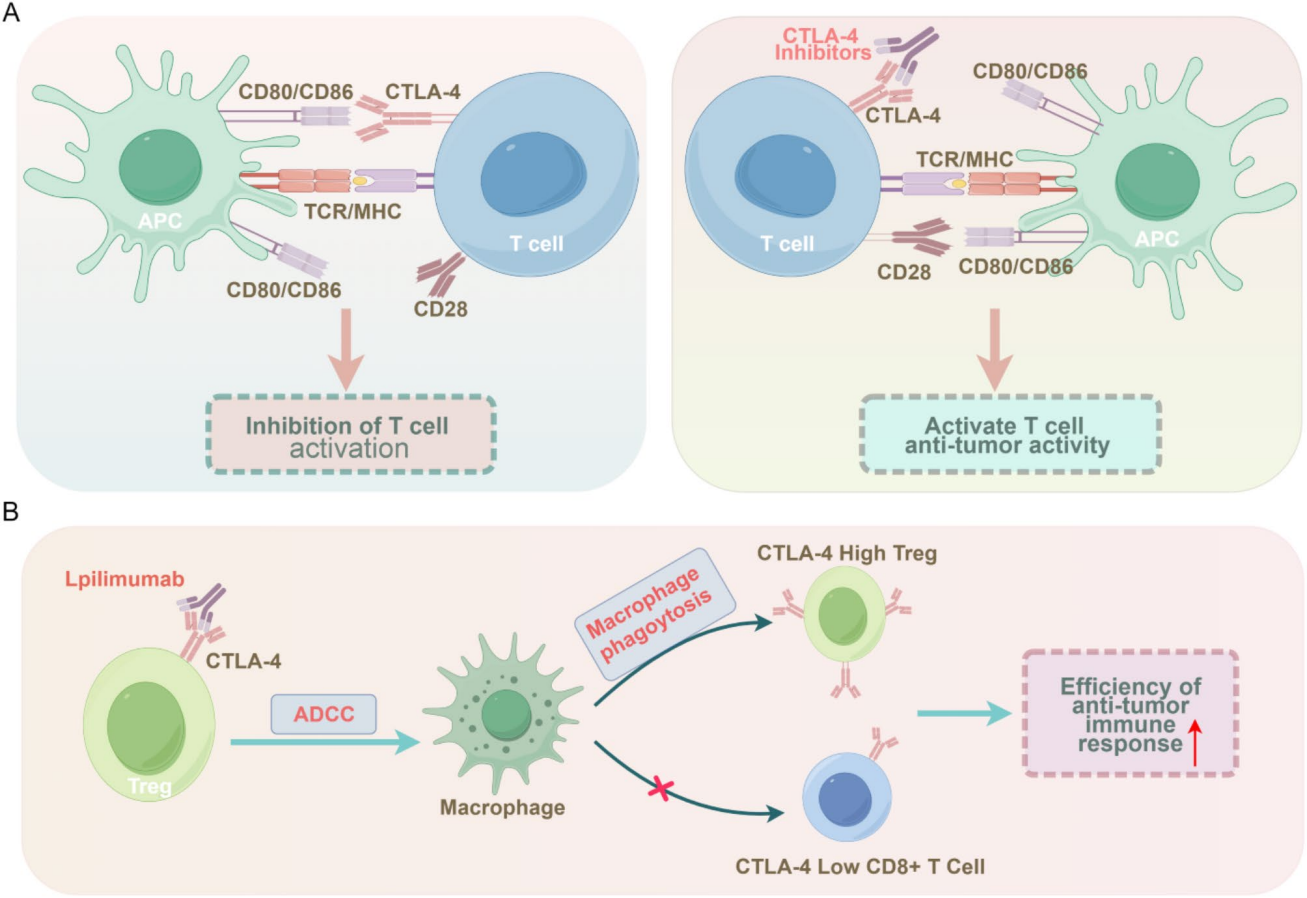
Immunological mechanisms mediated by CTLA-4. **(A)** When TCR-mediated costimulation binds to antigens presented by MHC on APCs, CTLA-4 preferentially binds to CD80/CD86, leading to the inhibition of T cell activity. When CTLA-4 inhibitors are introduced, CD80/CD86 binds to CD28 instead, reactivating T cell immune activity. **(B)** When CTLA-4 on the surface of Tregs binds to Ipilimumab, ADCC is induced, resulting in the phagocytosis of Tregs with high CTLA-4 expression by macrophages. This also leads to the retention of CD8 + T cells with low CTLA-4 expression. CTLA-4, cytotoxic T-lymphocyte–associated antigen 4; MHC, major histocompatibility complex; TCR, T-cell receptor; ADCC, antique-dependent cell-mediated cytotoxicity

CTLA-4, a key immunosuppressive checkpoint receptor, is primarily expressed on activated T cells [129]. More recent studies have also detected CTLA-4 expression on other immune cell types, such as regulatory T cells (Tregs) and exhausted T cells (Tex) [130]. The major histocompatibility complex (MHC) pathway underpins the immune system’s ability to recognize and respond to pathogens, with antigen presentation by tumor cells in the TME mainly occurring through the MHC I pathway [131]. B7 family proteins (CD80 and CD86) on antigen-presenting cells (APCs) bind to CD28 on T cells, activating them to target tumor cells [4, 5]. However, CTLA-4 on T cells exhibits a stronger affinity than CD28 and competes for binding to CD80/CD86, not only inhibiting T cell activation but also inducing immune tolerance and the formation of Tex [132, 133]. CTLA-4 inhibitors, such as Ipilimumab, alleviate the suppression of T cells by blocking the binding of CTLA-4 to CD80/CD86, thereby promoting T cell activation, proliferation, and effector function (Fig. 2A) [127, 128]. Additionally, research indicates that CTLA-4 on Treg cells can induce antibody-dependent cellular cytotoxicity (ADCC) when bound to Ipilimumab [134]. Macrophages in the TME recognize and phagocytose Tregs with high CTLA-4 expression [135, 136], whereas CD8 + effector T cells, with low CTLA-4 expression, remain unaffected [137, 138], further enhancing the efficiency of the anti-tumor immune response (Fig. 2B). A recent study also highlighted that CTLA-4 inhibitors promote the expansion of T helper 1 (Th1) cells and the formation of memory T cells during the early stages of the immune response [139].

### PD-1/PD-L1 inhibitors

PD-1, also known as CD279, is an inhibitory immune checkpoint receptor that is expressed on activated T cells, B cells, Tregs, natural killer (NK) cells, and macrophages. It plays a pivotal role in maintaining immune tolerance and preventing autoimmune diseases [140]. PD-1 binds to its ligands, PD-L1 and PD-L2, which are expressed on various somatic cells, including B cells, T cells, APCs, dendritic cells (DCs), and macrophages. This binding inhibits T cell activation and proliferation [140, 141], and additionally induces apoptosis of mature T cells while reducing apoptosis in Tregs [142].

**Fig. 3:**
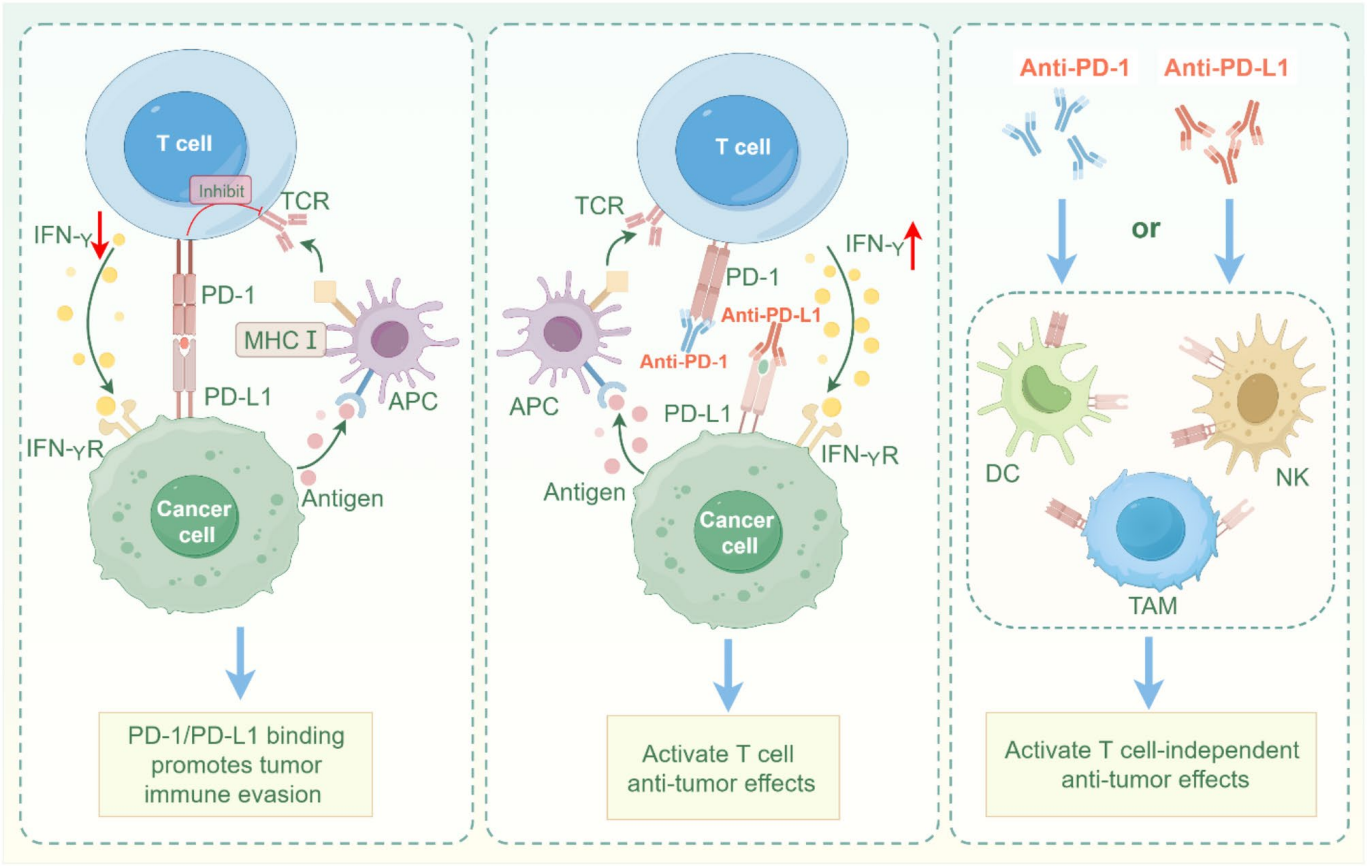
Immunological mechanisms mediated by PD-1/PD-L1. When TCR-mediated costimulation binds to antigens presented by MHC on APCs, PD-1 binding to PD-L1 inhibits T cell activity and reduces the secretion of IFN-γ by T cells. The use of PD-1/PD-L1 inhibitors reactivates T cell immune activity and increases IFN-γ release. In addition, PD-1/PD-L1 inhibitors can induce other immune cells to produce anti-tumor effects independent of T cells. TCR, T-cell receptor; MHC, major histocompatibility complex; PD-1, Programmed death receptor 1; DC, dendritic cells; NK, natural killer cell; TAM, Tumor-associated macrophages; IFN-γ, interferon gamma

Research has shown that PD-1 ligands, particularly PD-L1, are commonly expressed on tumor cells [140]. In the TME, T cells release interferon-gamma (IFN-γ) to kill tumor cells after recognizing abnormal antigens through the MHC I pathway [143]. However, IFN-γ’s role in the tumor immune response is often described as a “double-edged sword.” Some studies have suggested that PD-L1 is present on exosomes released by metastatic melanoma cells and that IFN-γ produced by CD8 + T cells can increase PD-L1 expression on these exosomes [143]. When PD-1 on T cells binds to PD-L1, the immune system mistakenly identifies tumor cells as normal, leading to T cell apoptosis and inhibition of T cell activation and proliferation [144, 145]. Since its clinical application, PD-1/PD-L1 inhibitors have shown remarkable efficacy in treating various cancers. The use of PD-1 or PD-L1 inhibitors blocks the interaction between PD-1 and PD-L1, which enhances the proliferation of CD8 + effector T cells and improves the local immune response against tumors. Moreover, these inhibitors can also relieve the inhibition of other immune cells, such as DCs and B cells, by the PD-1/PD-L1 pathway [146, 147]. This suggests that PD-1/PD-L1 inhibitors may also produce antitumor effects independent of T cells (Fig. 3). Additionally, recent studies have highlighted that Tcf1 + CD8 + T cells from tumor-draining lymph nodes (TDLNs) are the primary effector cells of PD-1 inhibitors, showing a high level of tumor cell inhibition [148, 149].

The role of biomarkers in predicting patient responses to PD-1/PD-L1 inhibitors is becoming increasingly recognized. This stems from the fact that, although PD-1 inhibitors exhibit significant efficacy in a small subset of cancer patients, their therapeutic benefits remain limited in the majority of cases [140, 141]. Notably, tumor cells may escape the antitumor effects of IFN-γ by silencing or mutating key molecules in the IFN-γ signaling pathway, such as JAK1/JAK2 and STATs. Such mutations often result in the loss of PD-L1 expression in tumor cells [143]. Research has shown that even in melanoma patients with a high tumor mutational burden (TMB)—a factor typically associated with better response to anti-PD-1/PD-L1 therapy—the expected benefits of PD-1 blockade may not be realized [150, 151]. Therefore, identifying reliable biomarkers is essential for accurately predicting which patients are most likely to benefit from this treatment modality.

### Discovery of novel immune checkpoints

The discovery of novel immune checkpoints has marked a significant advancement in the field of cancer immunotherapy, offering new targets and therapeutic strategies beyond traditional checkpoints like CTLA-4 and PD-1/PD-L1 [152]. Lymphocyte Activation Gene 3 (LAG-3) is one such checkpoint, primarily expressed on activated T cells, Tregs, and certain B cells [153]. LAG-3 inhibits T cell proliferation and cytokine secretion *via* the MHCII pathway. In 2022, the FDA approved the LAG-3 inhibitor, Relatlimab, in combination with nivolumab for the treatment of unresectable or metastatic melanoma in adults and children aged 12 or older, making LAG-3 the third immune checkpoint to be applied in clinical settings after PD-1 and CTLA-4 [153].

Recent studies have highlighted proteins such as TIM-3 (T cell immunoglobulin and mucin domain-3), VISTA (V-domain Ig suppressor of T cell activation), BTLA (B and T lymphocyte attenuator), CD47, and 4-1BB (CD137). However, the clinical application of these immune checkpoints remains in the exploratory stage [154–157]. TIM-3, which is mainly expressed on activated T cells, particularly those that are functionally exhausted, and Tregs, inhibits T cell function by binding to its ligands such as Galectin-9, CEACAM-1, and HMGB1, promoting immune escape in the TME [158]. Studies have shown that TIM-3 inhibitors can reverse T cell exhaustion and promote tumor regression in mice, thereby enhancing the antitumor effects of PD-1 inhibitors [159]. VISTA, expressed on activated T cells, Tregs, dendritic cells, and some tumor cells, inhibits T cell activity by binding to its ligand VSIG-3. The signaling pathway and biological function of VISTA remain under investigation, but its mechanism is believed to resemble that of PD-1 and CTLA-4 [160]. Preclinical studies have revealed that the concomitant administration of KVA12123, a novel VISTA-targeting agent, with anti-PD-1 immune checkpoint blockade exhibits synergistic antitumor activity [161]. BTLA, a single-chain transmembrane protein belonging to the immunoglobulin superfamily, is mainly expressed on B cells and T cells. By binding to its ligand HVEM (herpesvirus entry mediator), BTLA negatively regulates immune responses and suppresses T and B cell activity [155]. Studies have demonstrated that dual inhibition of BTLA and PD-1 significantly enhances the therapeutic efficacy of paclitaxel against intraperitoneally disseminated tumors [162]. CD47, another single-chain transmembrane protein, transmits the “Don’t Eat Me” signal. Many tumor cells express high levels of CD47, which binds to its ligand, SIRPα (signal regulatory protein alpha), inhibiting macrophage-mediated phagocytosis. This interaction enables tumor cells to evade immune surveillance and promotes tumor growth and metastasis [156]. Studies have shown that combining the blockade of CD47/SIRPα and PD-1/PD-L1 pathways is an effective intervention, restoring macrophage phagocytosis and producing substantial antitumor effects [163]. Unlike these inhibitory molecules, 4-1BB, a co-stimulatory molecule in the TNF (tumor necrosis factor) receptor superfamily, plays a critical role in T cell activation and proliferation, particularly in anti-tumor immunity. By binding to its ligand 4-1BBL, 4-1BB promotes T cell activation and the formation of memory T cells, playing a crucial role in enhancing the efficacy of PD-1/PD-L1 blockade [157]. Many studies suggest that combining novel immune checkpoint inhibitors with traditional ICIs (such as PD-1/PD-L1 and CTLA-4 inhibitors) could significantly improve cancer treatment outcomes and patient survival. These combinations, along with personalized treatment strategies, hold promise for more effective cancer immunotherapy [153].

### The exploration of combination therapy

Although ICIs have achieved remarkable success in cancer immunotherapy, their effectiveness is not universal, and they are not effective for all patients when used alone [8, 164]. As a result, the exploration of combination therapies has become a key area of research. In 2015, James Larkin et al. demonstrated for the first time that combining the CTLA-4 inhibitor Ipilimumab with the PD-1/PD-L1 inhibitor Nivolumab significantly extended the survival of patients with advanced melanoma [165]. This groundbreaking result marked the successful application of ICI combination therapy and set the stage for subsequent cancer immunotherapy research. Since then, increasing numbers of researchers have advocated for the combination of ICIs with other cancer therapies, showing promising clinical efficacy [166, 167]. For instance, Nivolumab combined with chemotherapy agents like Cisplatin and Gemcitabine has been used as a first-line treatment for urothelial carcinoma [168]. Moreover, studies suggest that combining ICIs with chemotherapy can induce ICD, activate the adaptive immune response, and reduce the suppression of tumor-infiltrating lymphocytes by the TME, thereby enhancing the effectiveness of immunotherapy [169]. The combination of ICIs with radiotherapy, targeted therapy, adoptive cell therapy, and the next generation of ICIs has also improved treatment outcomes and survival rates in patients [8, 152, 164]. Recently, some studies have highlighted the potential of combining ICIs with targeted therapies focused on RCD pathways—such as apoptosis, autophagy, ferroptosis, and disulfidptosis—as a promising approach for cancer treatment [170, 171]. RCD has been shown to play a pivotal role in carcinogenesis, and various forms of RCD can alter the TME by releasing pathogen- or damage-associated molecular patterns (PAMPs or DAMPs), which may enhance the efficacy of cancer treatments [172]. For example, Zhang et al. found that PD-1 signaling can inhibit the expression of PLPP1 (PD-1 signaling limits phospholipid phosphatase 1) in tumor-infiltrating CD8 + T cells *via* the Akt-GATA1 signaling pathway. This inhibition triggers ferroptosis in CD8 + T cells, thereby reducing their ability to combat tumors [173]. Yu et al. further demonstrated that PD-1/PD-L1 inhibitors can activate CD8 + T cells, inducing tumor cells to undergo various forms of RCD, such as ferroptosis, pyroptosis, and necroptosis [174]. These observations suggest that combining RCD inducers with ICIs could offer novel therapeutic strategies for cancer treatment by enhancing immune responses and overcoming drug resistance. This evidence supports the notion that targeting cell death signaling pathways in combination with ICI therapy may represent a promising approach to improving tumor treatment outcomes.

ICIs combination therapy has shown tremendous potential in cancer treatment. However, this approach also comes with a range of challenges and risks. One of the primary concerns is the increased occurrence of irAEs [175]. Different immune checkpoint inhibitors target the immune system through distinct mechanisms, and when used in combination, these effects may overlap, potentially leading to excessive immune responses and organ damage [1, 175]. Additionally, as combination therapies often amplify the side effects of individual drugs, the use of ICIs in combination may result in increased toxicity. For instance, when PD-1/PD-L1 inhibitors are combined with CTLA-4 inhibitors, patients may experience more severe side effects than when either drug is used alone, such as gastrointestinal reactions (e.g., diarrhea, colitis) and skin reactions (e.g., rashes, itching) [176, 177]. Furthermore, the side effects of combination therapy are often more complex, requiring more precise dose adjustments and treatment monitoring to prevent undue treatment burdens on patients. Therefore, to maximize therapeutic benefits while minimizing risks, future research needs to focus on developing accurate predictive tools and biomarkers, optimizing treatment regimens, and enhancing the management and monitoring of immune-related side effects.

## Disulfidptosis

Disulfidptosis represents a novel form of programmed cell death, distinct from traditional apoptosis, necroptosis, pyroptosis, autophagy, ferroptosis, and cuproptosis. It is characterized by the excessive accumulation of disulfides within cells that overexpress SLC7A11 under glucose starvation conditions, leading to disulfide stress and ultimately inducing cell death [19]. Studies have indicated that SLC7A11, a transporter protein responsible for the translocation of extracellular cysteine into cells [22], plays a pivotal role in the regulatory mechanisms of both ferroptosis and disulfidptosis [19, 24].

### Double-edged sword of the SLC7A11 in REDOX regulation

REDOX homeostasis is critical for cell survival [178]. Compared to normal cells, genetic mutations and metabolic reprogramming in cancer cells lead to increased reactive oxygen species (ROS) production within the TME, inducing oxidative stress [179, 180]. To survive and proliferate, cancer cells must maintain adequate GSH levels to neutralize excess ROS [181]. The Xc- system, a key regulator of GSH synthesis, consists of SLC3A2 and SLC7A11 dimers embedded in the cell membrane, with SLC7A11 serving as the functional subunit [182]. SLC7A11-mediated GSH synthesis is essential for cancer cell survival and antioxidant defense. Cystine is imported *via* SLC7A11, converted to cysteine in the cytoplasm, and contributes to GSH synthesis [183]. Glutathione peroxidase 4 (GPX4), utilizing GSH as a substrate, mitigates LPO, thereby preventing ferroptosis [184]. While SLC7A11 is typically associated with promoting cancer cell survival [22], under glucose starvation conditions, SLC7A11^high^ cancer cells exhibit a distinct cell death mechanism known as disulfidptosis [20, 185]. This results from excessive SLC7A11, which increases cystine uptake and reduction, heightening the cells’ sensitivity to disulfide stress [20]. Notably, under normal conditions, SLC7A11 generally suppresses ROS accumulation, but in the context of glucose deprivation, its overexpression exacerbates ROS production [185]. These observations highlight the pivotal role of SLC7A11 in regulating cell survival and death, suggesting that disulfidptosis could represent a novel therapeutic strategy for SLC7A11^high^ cancers.

Studies have shown that overexpression of SLC7A11 is closely associated with tumor cell resistance to chemotherapy and radiotherapy. Targeting SLC7A11 can enhance tumor cell sensitivity to treatment, overcome resistance, and improve therapeutic outcomes [34, 186]. However, current targeted therapies against SLC7A11 are still in the research phase. For example, researchers have revealed the potential of targeting SLC7A11 in pancreatic cancer treatment. They found that inhibiting SLC7A11 can reduce the generation of cancer-associated fibroblasts (CAFs), thereby decreasing tumor invasiveness and resistance [36]. Additionally, researchers discovered that SOCS2 induces the ubiquitination and degradation of SLC7A11, activating ferroptosis and enhancing the radiosensitivity of hepatocellular carcinoma [187]. These findings suggest that targeted therapies against SLC7A11 hold significant clinical potential in cancer treatment and warrant further research and clinical application.

#### SLC7A11 promotes anti-oxidation in cancer cells and inhibits ferroptosis

SLC7A11 is highly expressed in various human cancers, including non-small cell lung cancer, breast cancer, colorectal cancer, liver cancer, pancreatic cancer, and melanoma [24, 25]. Overexpression of SLC7A11 is closely associated with the initiation and progression of these cancers, and silencing SLC7A11 can significantly inhibit cancer cell growth and migration [24, 188, 189]. The primary mechanism involves SLC7A11’s role in cystine uptake, promoting intracellular cysteine and GSH production, thus counteracting oxidative stress and ferroptosis [24].

**Fig. 4:**
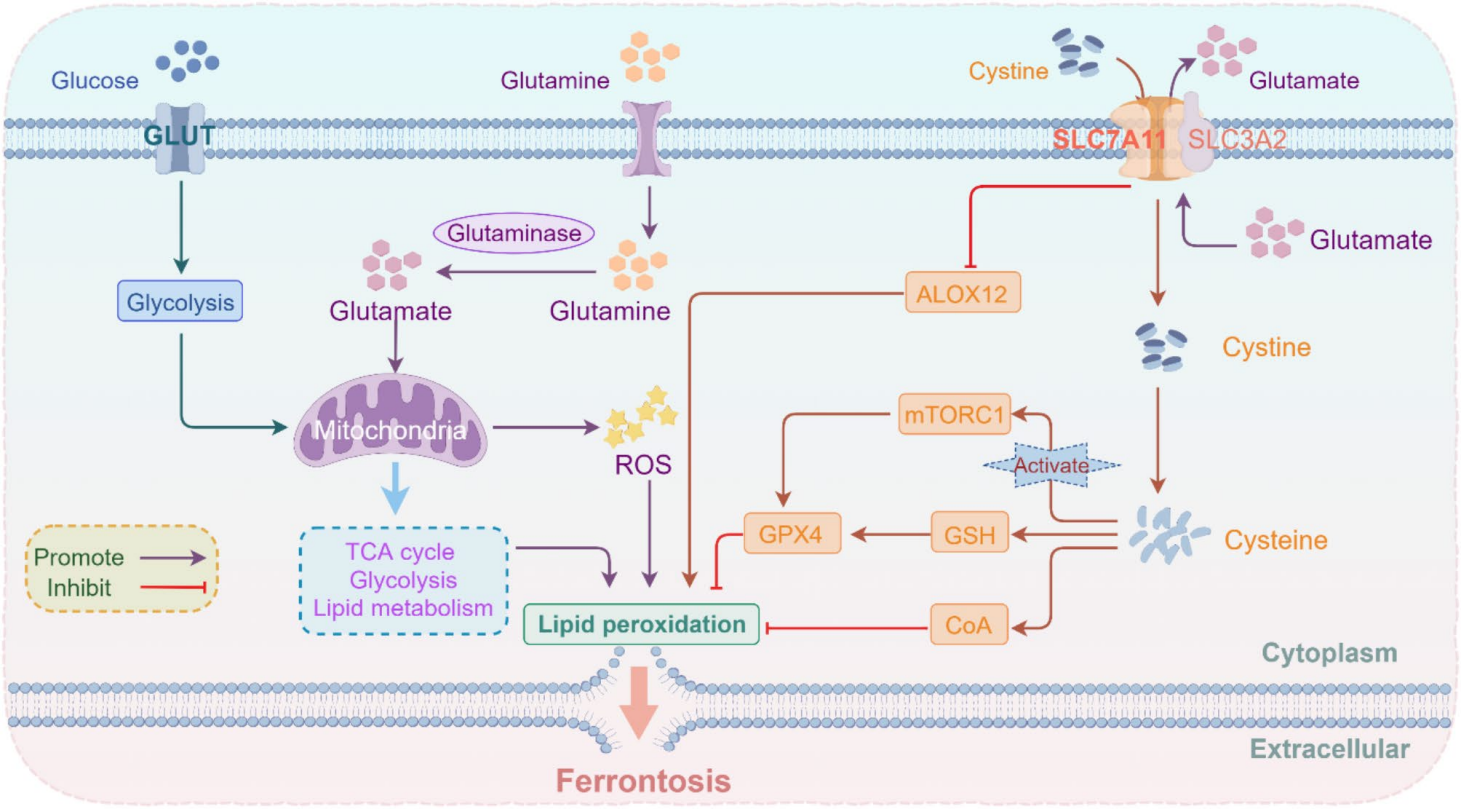
Mechanism of SLC7A11 inhibiting ferroptosis in cancer cells. In cancer cells, glutamine is converted to glutamate by glutaminase, which subsequently promotes the mitochondrial TCA cycle, glycolysis, mitochondrial ROS, and mitochondrial lipid metabolism, ultimately driving lipid peroxidation and ferroptosis. SLC7A11 inhibits ferroptosis by promoting cystine uptake and glutamate export, reducing lipid peroxidation through both GPX4-dependent and GPX4-independent pathways. TCA, Tricarboxylic acid; SLC7A11, recombinant solute carrier family 7, member 11; SLC3A2, solute carrier family 3 member 2; ROS, reactive oxygen species; GLUT, glucose transporter; GPX4, glutathione peroxidase 4; GSH, glutathione; ALOX12, arachidonate 12-lipoxygenase; mTORC1, mammalian target of rapamycin 1

Cancer cells, which require large amounts of GSH to mitigate oxidative stress, often face limitations in cysteine supply through the sulfur transfer and catabolic pathways. Consequently, most cancer cells acquire cysteine primarily from the extracellular environment [190]. However, cysteine is highly unstable outside the cell and typically exists in the oxidized dimer form, cystine. Therefore, cancer cells predominantly import cystine through SLC7A11, where it is subsequently reduced to cysteine in the cytoplasm [183] (Fig. 4).

Previous studies have demonstrated that SLC7A11 provides a survival advantage to cancer cells by inhibiting ferroptosis [184]. Some researchers have exploited cysteine proteases to consume extracellular cystine and cysteine in preclinical models, thereby inducing ferroptosis and inhibiting tumor progression [35]. In other studies, erastin and its analogs were used to downregulate SLC7A11 expression, also triggering ferroptosis in cancer cells [191]. Additionally, transcription factors such as p53 and BRCA1-associated protein 1 have been shown to inhibit SLC7A11 expression at the transcriptional level, promoting ferroptosis in cancer cells [48]. Several mechanisms regulating ferroptosis *via* SLC7A11 have been identified. Notably, the mTORC1 (mammalian target of rapamycin 1) signaling pathway has been implicated in the direct promotion of GPX4 synthesis [192]. Furthermore, SLC7A11 can inhibit ferroptosis through a GSH-independent mechanism. Research indicates that CoA (acetyl-CoA), synthesized from cysteine, can suppress ferroptosis by inhibiting LPO [193]. Given that SLC7A11 facilitates both cystine import and glutamate export, studies have found that inhibition of SLC7A11-mediated cystine uptake leads to elevated glutamate levels in the cell. This, in turn, promotes ferroptosis by enhancing iron-promoting activities within the mitochondria, including mitochondrial tricarboxylic acid cycling, glycolysis, ROS production, and lipid metabolism [194]. Intriguingly, SLC7A11 has also been shown to inhibit LPO and ferroptosis through direct interaction with ALOX12 (arachidonate 12-lipoxygenase) in a manner that does not rely on cystine transport [195, 196] (Fig. 4).

#### SLC7A11 promotes disufidptosis in glucose-deficient conditions

In the preceding section, the role of SLC7A11 in promoting antioxidant activity and inhibiting ferroptosis in cancer cells was discussed. However, for SLC7A11^high^ cancers, this “gain” comes with a significant trade-off. Specifically, because cystine import and glutamate export are coupled, SLC7A11^high^ cancer cells experience reduced glutamate levels. These cells rely heavily on glutamine production, catalyzed by GLS (glutaminase) in the mitochondria, to meet their survival requirements [22, 194]. Thus, targeting glutamine or glutaminase could serve as an effective therapeutic strategy for SLC7A11^high^ cancers. Studies have shown that glutamine deprivation or inhibition of glutaminase can suppress cell growth, with glutamine deficiency being linked to apoptosis [197].

**Fig. 5:**
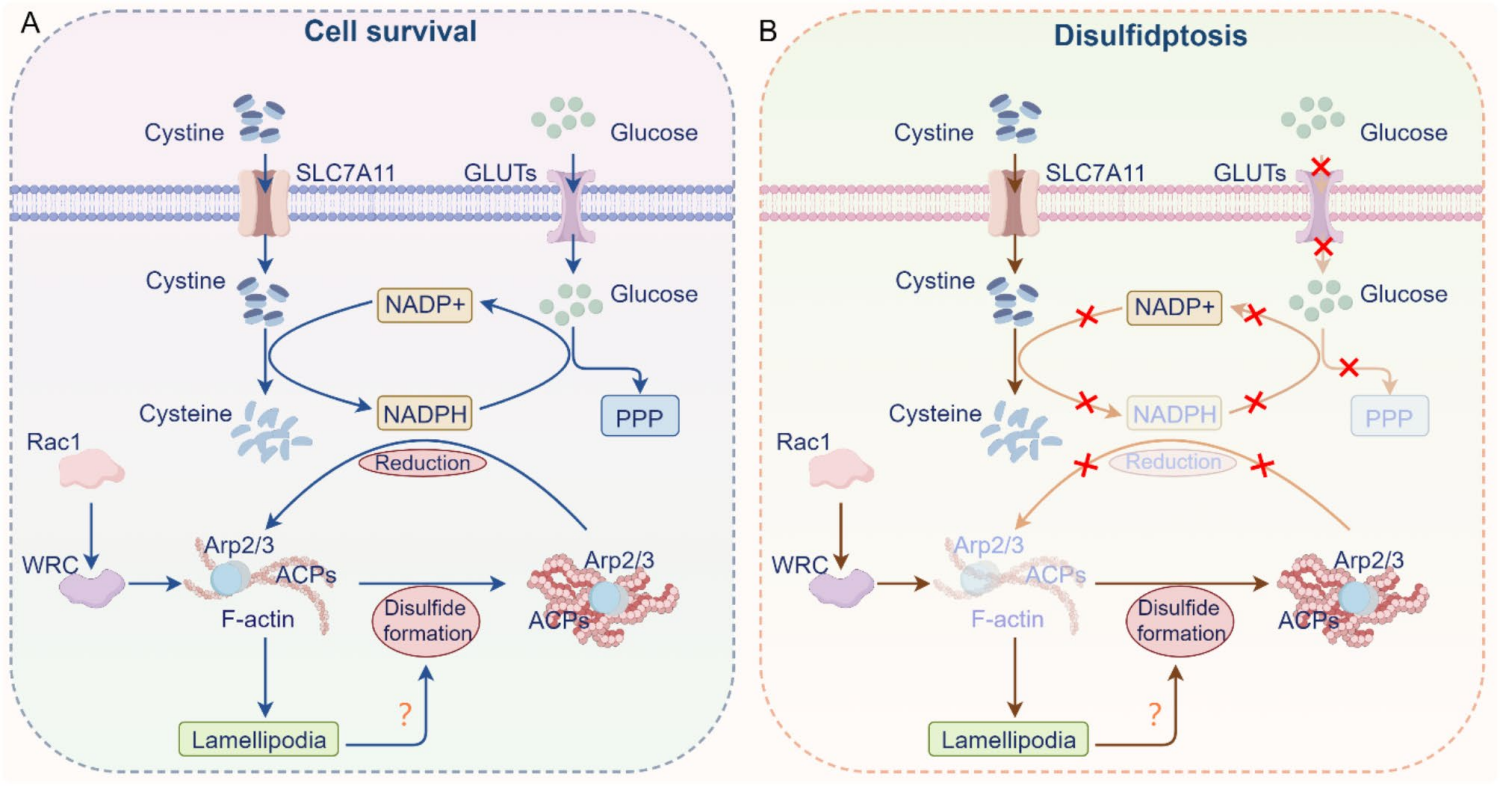
Mechanism of disulfidptosis regulation. **(A)** SLC7A11^high^ cells exhibit a high cystine uptake rate, which is subsequently reduced to cysteine by NADPH. NADPH is primarily derived from glucose *via* PPP. **(B)** In the absence of glucose, NADPH levels decrease, leading to the abnormal accumulation of cystine and other disulfide bond-containing molecules in SLC7A11^high^ cells. This accumulation promotes the formation of disulfide bonds within actin cytoskeletal proteins, ultimately resulting in the collapse of the actin network and initiating disulfidptosis. The Rac1-WRC-Arp2/3 pathway-mediated branched actin polymerization and lamellipodia formation may create a conducive environment for disulfide bond formation within actin proteins. PPP, pentose phosphate pathway; Rac1, Ras-related C3 botulinum toxin substrate 1; WRC, WAVE regulatory complex; GLUT, glucose transporter; NADPH, niacinamide adenine dinucleotide phosphate

Initially, it was believed that glucose deficiency-induced overexpression of SLC7A11 served as an adaptive response to maintain cell survival [198]. However, subsequent functional analyses revealed that SLC7A11^high^ cancer cells, being highly dependent on glucose, actually experience increased cell death under glucose deprivation [20]. Interestingly, inhibiting SLC7A11 expression under glucose-deficient conditions enhanced cell survival [19, 20]. This paradoxical effect is due to the high glucose dependence of SLC7A11^high^ cancer cells, which is intricately linked to cystine uptake and its reduction process [199]. Cystine, due to its low solubility and potential toxicity, needs to be rapidly converted to soluble cysteine *via* NADPH-dependent reduction [199]. NADPH is primarily provided by glucose metabolism through PPP, and in the absence of glucose, NADPH is quickly depleted. This depletion, in turn, leads to an accumulation of disulfide molecules, such as cystine, inducing disulfide stress and triggering disulfidptosis [199, 200] (Fig. 5A). Recent metabolomics and isotope tracer studies have further confirmed that high expression of SLC7A11 shifts intracellular glucose metabolism toward the PPP, increasing NADPH production [201, 202]. This underscores the critical dependence of SLC7A11^high^ cells on NADPH for their survival.

### Disulfide stress and disufidptosis

Cellular disulfide stress, characterized by NADPH depletion and abnormal accumulation of disulfide molecules, is a critical event in SLC7A11-high cancer cells [200]. A recent study under glucose starvation conditions coined this form of SLC7A11^high^ cancer cell death as “disulfidptosis,” a unique manifestation of cell death mediated by disulfide stress [20]. Research has indicated that disulfidptosis can be regulated by genes such as SLC7A11 or pharmacological agents like disulfide bond reducers and 2-deoxy-glucose [20, 203, 204]. Since disulfidptosis represents a disulfide stress-induced PCD, it is considered a novel form of RCD. While the precise mechanisms underlying this form of cell death are not fully understood, disulfidptosis appears to differ from other known RCDs. For instance, disulfidptosis does not exhibit typical hallmarks of other cell death mechanisms, such as ATP depletion or caspase-3 activation. Notably, inhibitors of other RCD pathways, such as ciclopirox olamine and ferroptosis inhibitors (e.g., Fer-1), or knockout of genes associated with ferroptosis or apoptosis, do not seem to affect disulfidptosis [20, 205]. Interestingly, while cancer cells with low SLC7A11 levels also undergo cell death under glucose deprivation, this form of death appears to be associated with apoptosis [20]. In summary, the characteristics of disufidptosis primarily include high expression of SLC7A11, depletion of NADPH, and atrophy of actin filaments (F-actin). These features distinctly differentiate disufidptosis from other forms of cell death.

Disulfide stress, as a specialized form of oxidative stress, induces cysteine residues (-SH) on redox-sensitive proteins to form mixed disulfide bonds with GSH, promoting the protein S-glutathionylation (SSG) reaction. This process is reversible and can be reversed by Glutaredoxin-1 (Grx1) [206, 207]. Under normal conditions, SSG primarily occurs in the endoplasmic reticulum and plays a role in regulating cell survival and function by modulating protein activities such as enzyme function, protein stability, and subcellular localization [208]. However, disulfide stress can lead to the excessive accumulation of disulfide bonds in cytoplasmic proteins, altering their original functions [209]. Consequently, the disulfidptosis observed in SLC7A11^high^ cells may be linked to functional abnormalities caused by the abnormal accumulation of disulfide bonds in specific proteins.

To further elucidate the protein regulation mechanisms underlying disulfidptosis, a proteomic study of SLC7A11^high^ cancer cells under glucose starvation revealed significant enrichment of actin cytoskeleton proteins, accompanied by the accumulation of disulfide bonds. This process promotes the contraction and detachment of F-actin from the plasma membrane, ultimately leading to disulfidptosis [209]. Moreover, genome-wide CRISPR screening of SLC7A11^high^ cancer cells identified that, in addition to the expected XC-system (SLC7A3 and SLC7A11), NCKAP1 (Nck-associated protein 1) also plays a role in promoting disulfidptosis [210]. As a key component of the WAVE regulatory complex (WRC), NCKAP1 does not affect cystine uptake or the NADP+/NADPH ratio but instead promotes glucose starvation-induced disulfide bond formation and actin cytoskeleton contraction [210, 211]. Further investigation revealed that the Rac1 (Ras-related C3 botulinum toxin substrate 1)-activated WRC stimulates the Arp2/3 (actin-related protein 2/3) complex, leading to the formation of lamellipodial pseudopodia and facilitating disulfidptosis [200, 212]. Additionally, the knockout of other WRC subunits was shown to inhibit disulfidptosis [200]. These observations suggest that the Rac1-WRC regulatory pathway promotes disulfidptosis through the mediation of actin polymerization and lamellipodial formation under disulfide stress (Fig. 5B).

In previous studies, proteins such as GAPDH (glyceraldehyde-3-phosphate dehydrogenase), Trx (thioredoxin), and Prxs (peroxiredoxins) have been implicated in the formation and breaking of disulfide bonds by regulating intracellular REDOX states [213–215]. The NF-κB and JNK signaling pathways are also believed to trigger disulfidptosis by modulating intracellular REDOX levels [20, 216]. Furthermore, recent studies suggest that downstream signaling molecules activated by tyrosine kinase receptors are closely associated with cell proliferation, survival, invasion, and migration. Under glucose deprivation, tyrosine kinase receptors are activated, indicating that tyrosine kinase signaling may be implicated in the early stages of disulfidptosis [217].

Based on the above studies, we propose several methods for identifying or detecting disulfidptosis in cells, including: (1) Measuring intracellular cystine content changes using commercial kits; (2) Assessing the expression of SLC7A11 via qPCR, WB (Western blotting), or immunofluorescence; (3) Analyzing the expression of the cytoskeletal protein Actin using immunofluorescence; (4) Performing mass spectrometry to examine the disulfide bond levels in cytoskeletal proteins; and (5) Detecting cell death rates using PI (propidium iodide) staining and flow cytometry.

## Research status of disulfidptosis in cancer treatment

SLC7A11 plays a critical role in regulating cell ferroptosis and apoptosis, promoting antioxidant defenses in cancer cells while inhibiting ferroptosis [20, 50, 218, 219]. Consequently, SLC7A11^high^ cancer cells are prone to developing resistance to ferroptosis and apoptosis inducers [220–223]. In such cases, targeting disulfidptosis-related pathways may offer a promising new approach for treating SLC7A11^high^ cancers. Despite the potential of these pathways to influence the clinical efficacy of ICIs, therapies specifically targeting cell disulfidptosis in cancer treatment remain under investigation (Table 3).

**Table 3:** Potential targets and drugs that promote cell disulfidptosis

Type	Target	Drug	Reference
**Inducer**	SLC7A11	Sulforaphane	[224]
		Curcumin	[225]
		Buthionine-Sulfoximine	[226]
		N-acetylcysteine	[183, 227]
		Pterostilbene	[228]
		Resveratrol	[229]
		Lactoferrin	[34]
		Vitamin D	[183, 230]
	Rac1-WRC	NSC23766	[231, 232]
		Ehop-016	[233, 234]
		EHT1864	[232]
		FMLP	[235]
		N-formylmethionine	[236]
	Oxidative Stress	Arsenic Trioxide	[237, 238]
		Paclitaxel	[239]
		Resveratrol	[240, 241]
		Curcumin	[242]
		Quercetin	[243]
		MPTP	[244]
		Melphalan	[245]
		Cyclophosphamide	[246, 247]
		Auranofin	[248]
		Fisetin	[249]
		Paraquat	[250]
		Tert-butyl hydroperoxide	[251]
		Doxorubicin	[252]
		Vincristine	[253, 254]
**Inhibitor**	GLUT	WZB117	[255]
		STF-31	[256]
		Bay-876	[257, 258]
		Phloretin	[259]
		Sitagliptin	[260]
		Phlorizin	[261]
		Berberine	[262, 263]
	TrxR	Auranofin	[264]
		PX-12	[265]
		TRI-1	[266]
		Arsenic Trioxide	[267]
		Chloramphenicol	[268]
		Ruthenium Complexes	[269]
		2-AAPA	[270–272]
	GR	Carmustine	[273]
		Auranofin	[274]
		2-AAPA	[275]
		Tert-butylhydroquinone	[276]
		Oxaliplatin	[277]
		Buthionine sulfoximine	[278]
		Dehydroepiandrosterone	[279]
		Schisandrin B	[280]
		Vitexin	[281]
	PDI	Epalrestat	[282, 283]
		Tunicamycin	[284, 285]
		Virodhamine	[286, 287]
		Allicin	[288]

SLC7A11, solute carrier family 7 member 11; GLUT, glucose transporter; TrxR, thioredoxin reductase; GR, glutathione reductase; PDI, protein disulfide isomeras; MPTP, 1-Methyl-4-phenyl-1,2,3,6-tetrahydropyridine; FMLP, formyl-met-leu-phe; TRI-1, thioredoxin reductase inhibitor 1; 2-AAPA, 2-aminophenyl arsenic acid

### Targeting SLC7A11high cancer cells

Several preclinical studies have explored strategies to inhibit the proliferation and metastasis of SLC7A11^high^ cancer cells by promoting disulfidptosis [289–291]. One such strategy involves inhibiting glucose transport or creating a glucose-deprived environment, which is essential for triggering disulfidptosis in SLC7A11^high^ cells [20, 292]. In a study focusing on metabolic interventions in cancer, researchers demonstrated that inhibiting GLUT (glucose transporter) could rapidly kill SLC7A11^high^ cancer cells, likely by inducing disulfidptosis [293]. This suggests that targeting disulfidptosis could hold significant therapeutic potential for treating metabolically vulnerable diseases, including SLC7A11^high^ cancers.

**Fig. 6:**
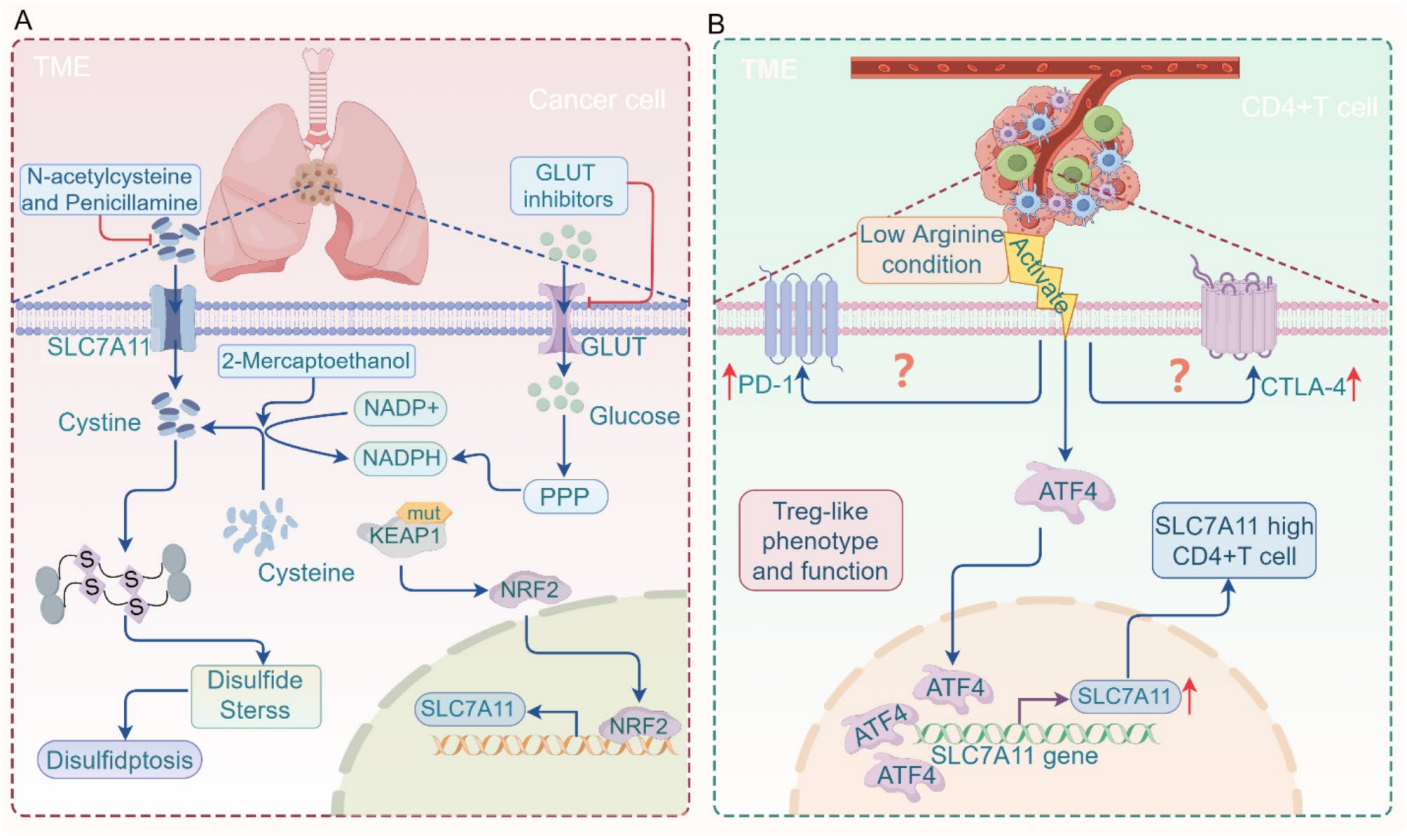
Research progress of disulfidptosis in the TME. **(A)** In SLC7A11^high^ lung cancer cells, KEAP1 mutation is a key factor leading to SLC7A11 overexpression. GLUT inhibitors can block the PPP, reducing NADPH production and promoting disulfidptosis. N-acetylcysteine and penicillamine can reduce cystine to cysteine extracellularly, or 2-mercaptoethanol can convert cysteine to cystine intracellularly, thereby restoring NADPH levels and inhibiting disulfidptosis. **(B)** In the TME, the low arginine environment caused by excessive tumor cell absorption activates ATF4 in CD4 + T cells, promoting SLC7A11 expression and inducing a transient Treg-like phenotype and function in CD4 + T cells. Furthermore, low arginine levels also enhance the expression of PD-1 and CTLA-4 in CD4 + T cells. KEAP1, kelch-like ECH-associated protein 1; PPP, pentose phosphate pathway; TME, tumor microenvironment; NRF2, nuclear factor-erythroid 2-related factor 2; ATF4, activating transcription factor-4; GLUT, glucose transporter; NADPH, niacinamide adenine dinucleotide phosphate; SLC7A11, recombinant solute carrier family 7

A typical example of SLC7A11^high^ cancer is KEAP1 (kelch-like ECH-associated protein 1)-mutant lung cancer [294, 295]. Under normal conditions, NRF2 (nuclear factor erythroid 2-related factor 2), a transcription factor sensitive to oxidative stress, binds to its negative regulatory protein KEAP1, leading to KEAP1-mediated NRF2 ubiquitination and degradation through the ubiquitin-proteasome system (UPS) [296, 297]. When KEAP1 is mutated or deleted, NRF2 is stabilized, resulting in increased transcription of SLC7A11 [295, 298]. Studies have shown that KEAP1 mutations or deletions enhance the glucose dependence of lung cancer cells, which, due to increased SLC7A11 expression, exhibit disulfidptosis characteristics under glucose-deprived conditions [293]. This further supports the idea that disulfidptosis in SLC7A11^high^ cancer cells can be promoted by inhibiting glucose transport. GLUT inhibitors, as a targeted therapeutic strategy, aim to suppress glucose uptake in tumor cells, thereby limiting their energy supply and inhibiting tumor growth. Recent advancements have led to the development of GLUT inhibitors (such as KL-11743, Fasentin, and Bay-876) that block glucose uptake in SLC7A11^high^ cancer cells [257, 299, 300]. Studies indicate that treatment with GLUT inhibitors disrupts the PPP, preventing NADPH production during glucose starvation. This results in a marked increase in the NADP+/NADPH ratio and the accumulation of disulfide bond molecules in cancer cells. Consequently, the integrity of the F-actin network is compromised, ultimately leading to disulfidptosis in SLC7A11^high^ cancer cells [293, 301] (Fig. 6A). However, as one of the most widely distributed glucose transporters in the human body, GLUT1 is not only overexpressed on cancer cell membranes but also exists in other normal cells [293, 301]. Therefore, GLUT1 inhibitors may produce certain side effects or off-target effects on normal cells. Studies have shown that the most common side effects of GLUT1 inhibitors include hypoglycemia and neurological reactions [302, 303]. Furthermore, the off-target effects of GLUT1 inhibitors may also affect glucose transport in intestinal epithelial cells, leading to gastrointestinal reactions such as diarrhea [303]. To mitigate these issues, researchers are developing spatio-temporally selective GLUT1 inhibitors (WZB117-PPG). These inhibitors can be released only upon reaching the target cells, thereby reducing their impact on normal cells [304].

Studies have shown that during osteolytic metastasis of SLC7A11-high cancers, such as lung and breast cancers, a reciprocal feedback loop between osteoclasts and tumor cells contributes to disease progression [305–308]. Recent research has revealed upregulation of SLC7A11 expression during osteoclast differentiation [309, 310]. Inhibition of TXNRD1 (thioredoxinreductase1) expression in osteoclast precursors results in cystine accumulation, leading to cell death [310]. Furthermore, moderate overexpression of SLC7A11 confers resistance to oxidative damage induced by peroxides (e.g., H_2_O_2_), while excessive peroxide exposure in SLC7A11-overexpressing cancer cells triggers disulfide stress, culminating in cell death [25]. These mechanisms are likely linked to the disulfidptosis pathway.

Strategies to manage disulfide stress primarily involve extracellular reduction of cystine to cysteine (e.g., n-acetylcysteine, penicillamine) [311, 312] or intracellular conversion of cysteine to cystine (e.g., 2-thioglycolic acid) [313–315], which restore NADPH levels and mitigate disulfidptosis in glucose-deprived SLC7A11^high^ cancer cells. Additionally, thiol oxidants (e.g., diamine, diethyl maleate) have been demonstrated to promote the abnormal accumulation of disulfide-bonded molecules (e.g., cystine, glutamylcystine), thereby inducing disulfidptosis in SLC7A11^high^ cells [20]. These observations underscore the potential of glucose starvation, TXNRD1 inhibition, and H_2_O_2_ treatment as conditions for inducing disulfidptosis. In clinical practice, oxidative stress inducers (such as Paclitaxel, Cyclophosphamide, and Doxorubicin) and glutathione reductase inhibitors (e.g., Oxaliplatin) have been widely employed in the treatment of various cancers, demonstrating consistent and robust clinical efficacy. While current research has not yet provided direct evidence that these chemotherapeutic agents can induce disulfidptosis, there is a compelling rationale for a potential association, as the molecular mechanisms regulated by these drugs appear to overlap with the pathways implicated in disulfidptosis. As the mechanism of disulfidptosis is further explored in both preclinical and clinical settings, precision medicine for SLC7A11^high^ cancers may become a feasible therapeutic strategy.

### Targeting SLC7A11high immune cells

Recent studies on targeting disulfidptosis-related pathways have primarily focused on SLC7A11^high^ cancer cells [20, 316]. In this “high-yield, high-risk” metabolic state, tumor cells can undergo disulfidptosis when exposed to glucose starvation [20]. However, whether disulfidptosis also occurs in immune cells remains unclear. Current evidence suggests that the primary conditions for triggering disulfidptosis include glucose starvation and high SLC7A11 expression [20]. Within the TME, the “infinite proliferation” characteristic of cancer cells drives nutrient competition, depriving other cells, including immune cells, of essential resources such as glucose and amino acids. This nutrient depletion is a key factor contributing to T cell dysfunction and exhaustion [317, 318]. Furthermore, cellular heterogeneity within the TME indicates the coexistence of SLC7A11-positive and SLC7A11-negative immune cells [319–321]. Although no detailed studies have confirmed this, it is conceivable that cancer cells may induce disulfidptosis in immune cells, potentially increasing the proportion of SLC7A11-positive immune cells.

In 2024, Zou et al. published a study in Cell Reports highlighting arginine, a conditionally essential amino acid with potent immunomodulatory properties, as a critical nutrient acquired by tumor cells in the TME. This acquisition occurs not only directly but also *via* argininosuccinate synthase (ASS), which converts citrulline to arginine, enabling tumor cells to resist arginine starvation [322]. While it is generally believed that T cells do not express ASS, researchers found that under arginine-starved conditions (~ 20 µM), CD4 + T cells can be induced to express ASS during early activation. This transient ASS expression confers Treg-like immunosuppressive properties to CD4 + T cells [322–324]. Mechanistic studies revealed that arginine depletion leads to high expression of ATF4 (activating transcription factor-4) in CD4 + T cells. Accumulated ATF4 binds to the promoter region of SLC7A11, accelerating its transcription and resulting in overexpression of SLC7A11 in CD4 + T cells [322] (Fig. 6B). These findings indirectly suggest that tumor cells might induce SLC7A11 overexpression in some T cells under specific TME conditions. However, whether SLC7A11^high^ immune cells are susceptible to disulfidptosis remains to be experimentally verified. Interestingly, under arginine-starved conditions, CD4 + T cells also exhibited high expression of immune checkpoint molecules such as CTLA-4 and PD-1 [322, 324]. This observation further supports the potential therapeutic strategy of targeting SLC7A11^high^ T cells in combination with ICIs for cancer treatment.

## ICIs and disulfidptosis

**Fig. 7:**
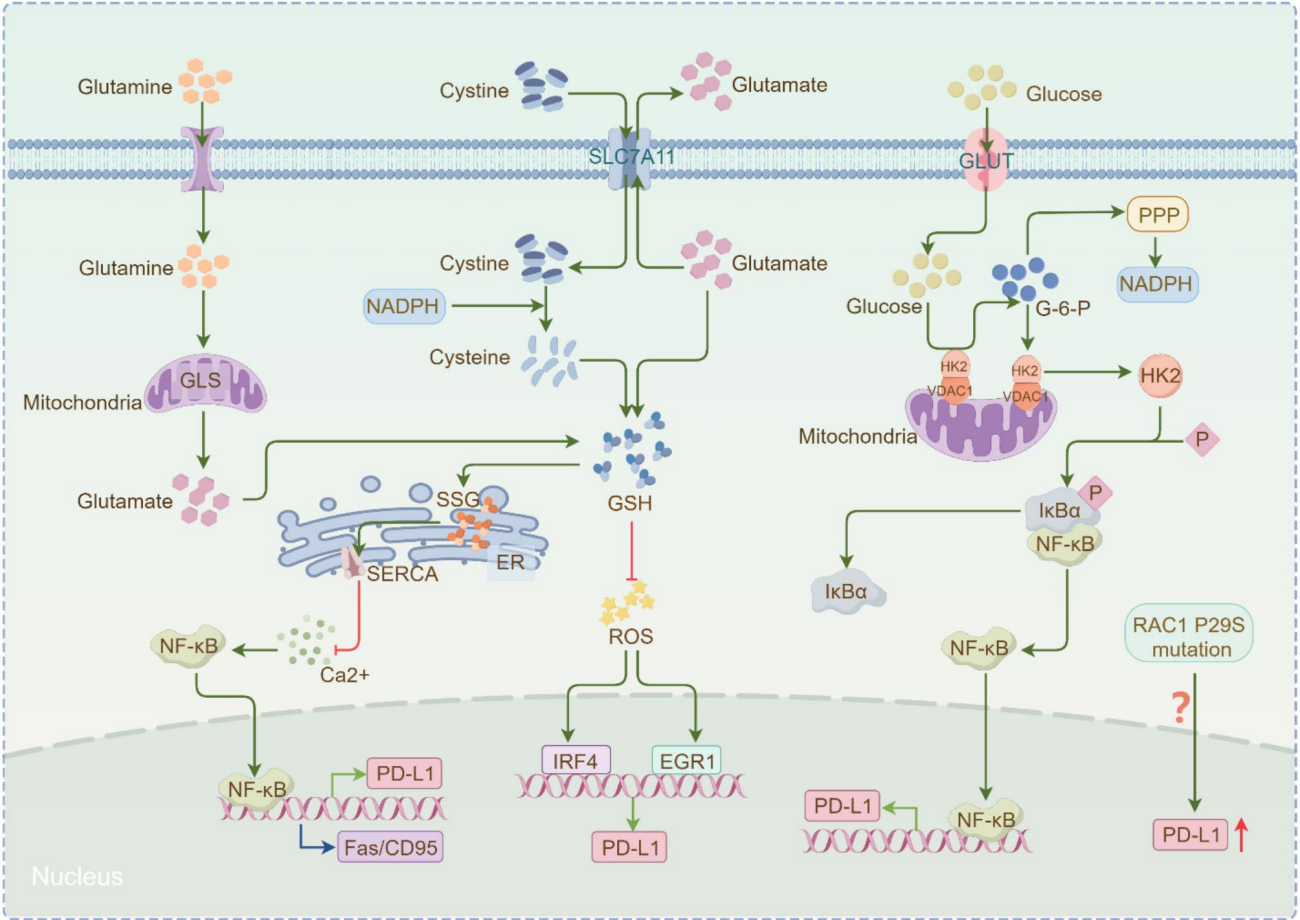
Relationship between disulfidptosis-related pathways and ICIs. SLC7A11 facilitates cystine uptake, which is reduced to cysteine by NADPH. The synthesis of GSH from cysteine and intracellular glutamate not only inhibits ROS-mediated PD-L1 expression but also regulates the SSG process in the endoplasmic reticulum, influencing the calcium/NF-κB signaling pathway by modulating SERCA activity. This ultimately suppresses PD-L1 and Fas/CD95 expression. Glucose also mediates the NF-κB signaling pathway, promoting PD-L1 expression. Additionally, the Rac1P29S mutation can increase PD-L1 expression. GSH, glutathione; SLC7A11, recombinant solute carrier family 7, member 11; ROS, reactive oxygen species; Rac1, Ras-related C3 botulinum toxin substrate 1; GLUT, glucose transporter; NADPH, niacinamide adenine dinucleotide phosphate; ER, endoplasmic reticulum; SERCA, sarcoendoplasmic reticulum calcium ATPase; SSG, protein S-glutathionylation; GLS, glutaminase

The clinical response of tumors to ICIs is influenced by numerous internal and external factors, with the heterogeneity of the TME identified as a key determinant [325, 326]. Within the TME, the disulfidptosis metabolic pathway predominantly involves the cysteamine metabolic pathway, PPP, and the Rac1-WRC pathway [171, 185]. As a result, disulfidptosis-related genes (DRGs) have been proposed as potential biomarkers for predicting tumor prognosis in ICI therapy [327, 328]. The combination of targeting DRGs and ICIs may represent a novel therapeutic strategy for SLC7A11^high^ cancers (Fig. 7).

### Cystine metabolic pathway

As previously discussed, SLC7A11 plays a pivotal role in facilitating the death of disulfide bonds and is integral to cysteamine metabolism. To maintain oxidative homeostasis and promote tumor progression, cancer cells upregulate the cystine/glutamate transport through SLC7A11 [329, 330]. This metabolic shift is further accelerated in SLC7A11^high^ tumor cells [330]. In a recent preclinical study on liver cancer, researchers found that Mer proto-oncogene tyrosine kinase (MerTK) enhances SLC7A11 expression *via* the ERK/SP1 signaling pathway. Simultaneously, it promotes the secretion of granulocyte colony-stimulating factor (G-CSF) by tumor cells, which increases the infiltration of MDSCs, thereby limiting ferroptosis and contributing to resistance to PD-1/PD-L1 blockade [27]. This suggests that targeting the ferroptosis-related pathway may no longer be the optimal solution in SLC7A11^high^ cancers. However, in a melanoma immunotherapy study, inhibiting SLC7A11 expression on tumor cells led to upregulation of intracellular transcription factors such as IRF4 (interferon regulatory factor 4) and EGR1 (early growth response protein 1), which in turn increased PD-L1 expression and significantly reduced the efficacy of PD-1/PD-L1 inhibitors [189]. Furthermore, melanoma cells can induce the polarization of M2 macrophages through the release of exosomes containing PD-L1, further diminishing the therapeutic effect of PD-1/PD-L1 inhibitors [189]. This suggests a positive correlation between SLC7A11 expression and the therapeutic efficacy of PD-1/PD-L1 inhibitors. However, the relationship between SLC7A11-mediated cell disulfidptosis and ICI therapy remains largely unexplored and warrants further investigation.

Upon entry into the cytoplasm *via* SLC7A11, cystine is converted into cysteine, which serves as a precursor for GSH, a critical antioxidant involved in cellular defense against oxidative stress [183, 192]. However, when glucose deficiency induces disulfidptosis, it is often accompanied by NADPH depletion, which inhibits the conversion of cysteine into cysteine and drastically reduces GSH synthesis efficiency [25, 331]. Additionally, SLC7A11 not only facilitates cystine uptake but also exports glutamate, making SLC7A11^high^ cancer cells highly dependent on glutamate produced from glutamine metabolism in the mitochondria [332, 333]. Studies have shown that inhibition of glutamine metabolism leads to decreased intracellular GSH levels, which in turn reduces SERCA (sarcoendoplasmic reticulum calcium ATPase) activity, activating the calcium/NF-κB signaling pathway and ultimately upregulating PD-L1 expression in tumor cells [334]. Although this theoretically impairs effector T cell activity, for primary drug-resistant tumor cells with low PD-L1 and Fas/CD95 expression, inhibiting glutamine metabolism alongside anti-PD-L1 therapy can significantly enhance T cell antitumor activity [334].

### Pentose phosphate pathway

PPP is a critical glucose catabolic pathway. In this process, glucose is converted to glucose-6-phosphate (G6P) under the action of hexokinase (HK), initiating the PPP [19, 335]. The PPP consists of two stages: the oxidation and non-oxidation stages, with NADPH primarily generated in the oxidation phase [19, 335]. It has been demonstrated in previous studies that SLC7A11-mediated cystine uptake in SLC7A11^high^ cancer cells consumes substantial amounts of NADPH, making these cells highly reliant on the PPP. When intracellular NADPH is depleted, disulfidptosis occurs [19, 336].

HK, a key kinase in glycolysis, regulates the glycolytic process by interacting with the mitochondrial outer membrane through its binding to voltage-dependent anion channels (VDAC) [337, 338]. In a high glucose environment, the HK subtype, such as HK2, dissociates from the mitochondria and binds to the T291 site of IκBα, where it phosphorylates IκBα [339]. This interaction promotes the binding of IκBα to µ-calpsin, leading to the degradation of IκBα and subsequent activation of NF-κB. The upregulation of NF-κB promotes the high expression of PD-L1 in tumor cells, contributing to immune evasion [339, 340]. In 2022, Guo et al. found that knocking out the IκBαT291 site in glioblastoma multiforme (GBM) cells inhibited the high glucose-induced expression of PD-L1 and promoted CD8 + T cell activation and infiltration. Furthermore, the therapeutic efficacy of PD-L1 inhibitors was significantly enhanced [339]. Additionally, combining HK inhibitors with PD-1 inhibitors showed stronger tumor inhibition [341, 342]. In the previous discussion, inhibition of the glycolytic pathway through glucose depletion was identified as a critical precondition for disulfidptosis. The deletion of the IκBαT291 site and the use of HK2 inhibitors directly or indirectly blocked the glycolytic process [339]. These findings suggest that targeting DRGs in combination with PD-L1 inhibitors could be a promising therapeutic strategy for treating SLC7A11^high^ cancers.

Although targeting the PPP in combination with ICIs may have synergistic effects, it also presents potential risks. Research suggests that inhibiting PPP can not only enhance immune cell activity but also lead to excessive immune activation. When combined with ICI therapy, this heightened immune response may trigger immune-related adverse effects, such as hepatitis, pneumonitis, and colitis [343–345]. Moreover, prolonged immune system stimulation could result in dysregulation and metabolic imbalances, potentially leading to immune tolerance and compromising long-term treatment efficacy [344, 345]. Additionally, while PPP-targeted therapy primarily affects tumor cells or specific immune cells, it may also impact other metabolically active cells, such as endothelial cells [335]. This off-target effect could introduce unforeseen complications during treatment. Therefore, the identification of precise biomarkers and the development of personalized therapeutic strategies are crucial to ensuring the success of this combination approach.

A study on triple-negative breast cancer (TNBC) highlighted the significant effect of radiation therapy (RT) on both the TME and systemic immunity [345]. RT induces oxidative stress, which upregulates the PPP and promotes NADPH production in tumor cells [345]. In the TME, high NADPH levels can not only downregulate PD-L1 expression through silencing of the ATM/ATR/Chk1 signaling pathways but also inhibit ICD, promoting tumor cell immune escape [345–347]. In tumor-associated macrophages (TAMs), excessive NADPH levels can drive polarization toward M1 macrophages and inhibit cancer progression [348]. To address this issue, in 2022, Wang et al. developed a multifunctional copolymer, BMS202@HZPNPs, which selectively depletes NADPH in tumor cells by targeting BMS202, a small molecule antagonist of PD-1/PD-L1. This strategy transforms “cold tumors” into “hot tumors” and enhances the response to anti-PD-L1 therapy [345, 349]. Furthermore, BMS202@HZPNPs can reprogram the immune metabolism of the TME to reduce its immunosuppressive effects [345]. These findings suggest that selectively eliminating NADPH in SLC7A11^high^ tumor cells in combination with anti-PD-L1 therapy could be an effective cancer treatment strategy. However, while disulfidptosis is strongly associated with NADPH depletion, the relationship between this drug and disulfidptosis in tumor cells remains to be further explored.

### Rac1-WRC pathway

Over the past few decades, the Rac1-WRC pathway has been implicated in a variety of biological processes, such as cytoskeletal recombination, protein kinase activation, cell proliferation, and migration [212, 350, 351]. Recent research has identified disulfide stress as a key factor that induces Rac1 overexpression, which, in turn, facilitates the assembly of the five subunits of the WRC-CYFIP1, NCKAP1, Abi2, WAVE2, and HSPC300-into a protein heteropentamer. This assembly activates the Arp2/3 complex, which regulates the reorganization of the actin cytoskeleton, promoting the formation of lamellipodial protrusions and ultimately leading to cell disulfidptosis [200, 350, 352, 353].

Although a direct regulatory relationship between Rac1 and immune checkpoint expression has not been definitively established, some studies suggest a potential connection. In 2015, researchers detected the P29S mutation of Rac1 in melanoma samples and observed that PD-L1 was selectively upregulated when Rac1^P29S^ was expressed, while it was downregulated when Rac1^P29S^ was depleted [354, 355]. Clinical trials revealed that patients with the Rac1^P29S^ mutation achieved higher clinical response rates and better outcomes when treated with PD-1 or PD-L1 inhibitors [354]. More recently, a study exploring the Rac1^P29S^ mutation in melanoma cells proposed that cyclin-dependent kinase 9 (CDK9) plays a significant regulatory role in Rac1^P29S^ activity [356]. In vitro studies demonstrated that inhibiting CDK9 expression not only hindered the proliferation of Rac1^P29S^ melanoma cells but also upregulated the surface expression of PD-L1 and MHC I proteins on tumor cells [356]. In vivo experiments further revealed that combining a CDK9 inhibitor with a PD-1 inhibitor significantly suppressed the growth of Rac1^P29S^ mutant melanoma [356]. These findings suggest that targeting Rac1 in combination with ICIs could be a promising strategy for tumor immunotherapy.

Additionally, WRC and Arp2/3, as downstream targets of the Rac1-WRC pathway during cell disulfidptosis, have been rarely linked to immune checkpoint regulation. However, a 2023 study by Claire’s team investigating the inhibitory mechanism of PD-1 and PD-L1 discovered an unexpected finding. They showed that PD-1 could inhibit T cell actin remodeling at immune synapses independently of its signaling motif. This inhibition was characterized by the absence of the Arp2/3 complex, which guides the formation of branched actin, as well as the dense distal lamellar reticular network of F-actin [357]. This suggests a potential link between PD-1 and the actin cytoskeleton dynamics of the Arp2/3 complex.

## Conclusion and prospect

Currently, the use of ICIs in cancer treatment is limited, with less than 30% of patients showing a positive clinical response [167, 358]. One of the primary challenges in tumor immunotherapy today is converting “cold tumors”—those with low clinical response rates—into “hot tumors” that are more responsive to immune treatments. While the discovery of new immune checkpoints and the development of novel ICIs are part of the solution, these alone are not sufficient [359, 360]. In addition to single-agent therapies, there is a growing consensus among researchers that the combination of ICIs with other therapeutic approaches may not only improve the clinical response rate but also reduce the resistance of tumor cells to ICIs to some extent [164, 361, 362]. This is particularly relevant for cancers characterized by high expression of SLC7A11, which is closely associated with tumor immune escape due to its role in promoting cell survival and antioxidant defense [363, 364]. Disulfidptosis represents a unique cell death mechanism in SLC7A11high tumors, and targeting pathways involved in disulfidptosis in conjunction with ICIs could offer a novel therapeutic strategy for the treatment of SLC7A11^high^ cancers.

This review summarizes the current research progress regarding ICIs in tumor immunotherapy, emphasizing the importance of combination therapies. It describes in detail the mechanism of disulfidptosis in SLC7A11^high^ tumor cells and explores the potential regulatory effects of disulfidptosis-related pathways on immune checkpoint expression. These findings collectively suggest that the efficacy of ICIs is significantly enhanced when tumor cells undergo disulfidptosis. However, despite progress in the study of disulfidptosis as a novel mechanism of tumor cell death, its combination with ICIs still faces certain clinical challenges. The following key issues warrant further exploration to fully realize the potential of this combined therapeutic approach.

First, glucose deficiency is an essential environmental factor for SLC7A11^high^ cells to undergo disulfidptosis. In vitro, this condition can be achieved by inhibiting glucose transporters (such as GLUT) or by creating a glucose-deprived culture environment. However, the in vivo situation is more complex, as it involves not only tumor cells but also various normal cells, including immune cells, within the TME. Therefore, identifying and targeting SLC7A11^high^ tumor cells specifically is essential for achieving therapeutic outcomes. Second, because tumor cells have high energy demands, they can induce a glucose starvation effect in immune cells within the TME. It is important to further investigate whether tumor cells can induce SLC7A11^high^ immune cells to undergo disulfidptosis, resulting in a “cold tumor” phenotype and potentially reducing the response rate to immunotherapy. Lastly, while cell disulfidptosis represents a novel non-apoptotic RCD mechanism that has only recently been proposed, its underlying mechanisms remain incompletely understood. Although targeting disulfidptosis-related pathways in combination with ICIs has shown positive results in cancer therapy, the precise relationship between disulfidptosis and immune checkpoint regulation requires further investigation.

In conclusion, promoting disulfidptosis in tumor cells is theoretically a feasible approach to improve the efficacy of ICIs. However, there are currently no in vivo or in vitro experiments to verify this hypothesis, let alone clinical studies to support it. Therefore, it is essential to continue exploring the immunological mechanisms underlying this treatment strategy in future research on SLC7A11^high^ tumor immunotherapy. Such studies will provide the necessary evidence for further in-depth research and ultimately benefit more cancer individuals.

## Data Availability

No datasets were generated or analysed during the current study.

## Abbreviations

SLC7A11: Solute carrier family 7 member 11
FDA: Food and Drug Administration
ORR: Objective response rate
irAEs: immune-related adverse events
GLUT: Glucose transporter
TrxR: Thioredoxin reductase
GR: Glutathione reductase
PDI: Protein disulfide isomeras
MPTP: 1-Methyl-4-phenyl-1,2,3,6-tetrahydropyridine
FMLP: Formyl-met-leu-phe
TRI: 1-Thioredoxin reductase inhibitor 1
2: AAPA-2-aminophenyl arsenic acid
CTLA: 4-Cytotoxic t-lymphocyte antigen 4
PD: 1-Programmed cell death protein 1
PD: L1-Programmed cell death ligand 1
LAG: 3-Lymphocyte activation gene 3
TIM: 3-T-cell immunoglobulin and mucin domain 3
TIGIT: T-cell immunoreceptor with Ig and ITIM domain
VISTA: V-domain ig suppressor of T cell activation
BTLA: B and T lymphocyte attenuator
OX40: Oxygen-40
4: 1BB-tumor necrosis factor receptor superfamily member 9
GITR: Glucocorticoid-induced tnfr-related gene
KRAS: Kirsten rat sarcoma viral oncogene homolog
Nrf2: Nuclear factor erythroid 2-related factor 2
HIF: 1α-Hypoxia-inducible factor 1-alpha
c: Myc-cellular myelocytomatosis oncogene
MAPK: Mitogen-activated protein kinase
BRAF: B-Raf proto-oncogene-serine/threonine kinase
SP1: Specificity protein 1
KEAP1: Kelch-like ECH-associated protein 1
ATF4: Activating transcription factor 4
NF: κB-Nuclear factor kappa-light-chain-enhancer of activated B cells
